# Role of natural transformation in the evolution of small cryptic plasmids in *Synechocystis* sp. PCC 6803

**DOI:** 10.1111/1758-2229.13203

**Published:** 2023-10-04

**Authors:** Fabian Nies, Tanita Wein, Dustin M. Hanke, Benjamin L. Springstein, Jaime Alcorta, Claudia Taubenheim, Tal Dagan

**Affiliations:** ^1^ Institute of General Microbiology Kiel University Kiel Germany; ^2^ Department of Molecular Genetics and Microbiology, Biological Sciences Faculty Pontifical Catholic University of Chile Santiago Chile; ^3^ Present address: Department of Molecular Genetics Weizmann Institute of Science Rehovot Israel; ^4^ Present address: Institute of Science and Technology Austria Klosterneuburg Austria; ^5^ Present address: Department of Internal Medicine II University Medical Center Schleswig‐Holstein Kiel Germany

## Abstract

Small cryptic plasmids have no clear effect on the host fitness and their functional repertoire remains obscure. The naturally competent cyanobacterium *Synechocystis* sp. PCC 6803 harbours several small cryptic plasmids; whether their evolution with this species is supported by horizontal transfer remains understudied. Here, we show that the small cryptic plasmid DNA is transferred in the population exclusively by natural transformation, where the transfer frequency of plasmid‐encoded genes is similar to that of chromosome‐encoded genes. Establishing a system to follow gene transfer, we compared the transfer frequency of genes encoded in cryptic plasmids pCA2.4 (2378 bp) and pCB2.4 (2345 bp) within and between populations of two *Synechocystis* sp. PCC 6803 labtypes (termed Kiel and Sevilla). Our results reveal that plasmid gene transfer frequency depends on the recipient labtype. Furthermore, gene transfer via whole plasmid uptake in the Sevilla labtype ranged among the lowest detected transfer rates in our experiments. Our study indicates that horizontal DNA transfer via natural transformation is frequent in the evolution of small cryptic plasmids that reside in naturally competent organisms. Furthermore, we suggest that the contribution of natural transformation to cryptic plasmid persistence in *Synechocystis* is limited.

## Graphical abstract

Establishing a system to follow gene transfer in *Synechocystis* sp. PCC 6803, we find that small cryptic plasmid DNA is transferred exclusively by natural transformation. Furthermore, we show that the transfer frequency of plasmid‐encoded and chromosome‐encoded genes is similar, while whole plasmid transfer is rare. We conclude that similarly to chromosomes, the evolution of plasmid genomes in naturally competent bacteria is accompanied by horizontal DNA transfer

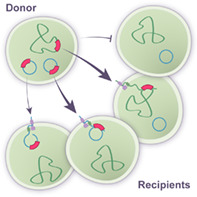

## INTRODUCTION

The size of plasmid genomes ranges over three orders of magnitude. Large plasmids encoding hundreds of genes are often essential for their host lifestyle (e.g., as in *Rhizobia*; Poole et al., 2018). Such large plasmids are non‐mobile and constitute an integral part of their host genome in the form of chromids (Harrison et al., 2010). In contrast, small plasmids encoding 10 genes or less are typically cryptic, that is, their utility to the host—if any—remains unknown (Novick et al., 1976). Notwithstanding, small plasmids in several genera have been reported to encode for antibiotic resistance genes, for example, in *Klebsiella* (Ramirez et al., 2019) or *Salmonella* (Tran et al., 2012), hence they might be beneficial for their host in the presence of antibiotics. Furthermore, small plasmids may encode for homologues to known toxin–antitoxin systems, as reported, for example, in *Aeromonas* plasmids (Pérez‐García et al., 2021). The presence of host‐addiction mechanisms could facilitate the plasmid persistence in the host population, also in the absence of plasmid‐encoded functions that are beneficial to the host. Another route to plasmid persistence in the population is plasmid horizontal transfer, which may compensate for unstable plasmid inheritance. Indeed, small cryptic plasmids often harbour a genetic repertoire that enables their mobilisation by conjugative plasmids residing in the same host (e.g., Barry et al., 2019; Dunn et al., 2005). Furthermore, small plasmids have been documented to be transferred via generalized transduction (e.g., in *Yersinia*; Hertwig et al., 1999) and natural transformation (e.g., in *Acinetobacter calcoaceticus*; Rochelle et al., 1988). The presence of plasmids in outer membrane vesicles (OMVs) furthermore led to suggestions that plasmids may be transferred by vesicles (e.g., in *E. coli*; Tran & Boedicker, 2019).

*Synechocystis* sp. PCC 6803 (hereafter *Synechocystis*) is a unicellular, freshwater cyanobacterium, which is naturally competent (Grigorieva & Shestakov, 1982). It is well characterized and is frequently used as a model strain to address basic and applied research questions in cyanobacteria. The transformation rate in *Synechocystis* is rather stable during exponential growth phase (Zang et al., 2007). The frequent use of this strain since its isolation in 1968 (Stanier et al., 1971) resulted in divergence from the original isolate into several substrains (Ikeuchi & Tabata, 2001; Kanesaki et al., 2012; Trautmann et al., 2012). These substrains show different phenotypes, such as motility or tolerance to glucose (Ikeuchi & Tabata, 2001) as well as stress sensitivity (Zavřel et al., 2017). *Synechocystis* harbours four large plasmids pSYSA (100,749 bp), pSYSG (44,343 bp), pSYSM (118,712 bp), and pSYSX (106,004 bp), and three small plasmids pCA2.4 (2378 bp), pCB2.4 (2345 bp), and pCC5.2 (5214 bp) (Castets et al., 1986; Chauvat et al., 1986; Kaneko et al., 2003; Xu & McFadden, 1997; Yang & McFadden, 1993, 1994). The three small plasmids of *Synechocystis* are all considered to replicate by a rolling circle mechanism (Xu & McFadden, 1997; Yang & McFadden, 1993, 1994). *Synechocystis* is polyploid and its chromosome copy number varies depending on the growth phase (see recent summary in (Nagy et al., 2021)). The small plasmid copy number relative to the chromosome varies between 0.4 and 7.4, depending on the growth phase and between autotrophic and mixotrophic growth; pCA2.4 and pCC5.2 are present in higher copy numbers compared to pCB2.4 (Berla & Pakrasi, 2012). Whether any of the *Synechocystis* small plasmids encodes for a function that is beneficial for the host remains unknown.

The small plasmid composition varies among the *Synechocystis* substrains. Indeed, absence and loss of the small cryptic plasmids in *Synechocystis* have been documented. For example, pCC5.2 loss has been reported but had no apparent effect on *Synechocystis* phenotype (Castets et al., 1986). While *Synechocystis* sp. PCC 6803GT (glucose tolerant substrain) contains all three small plasmids, *Synechocystis* sp. PCC 6803M (motile substrain) harbours only pCA2.4 (Jin et al., 2018). Furthermore, the small plasmids (but not the large plasmids) can be cured by CRISPR/Cas without any effect on the host phenotype (Xiao et al., 2018), hence the small plasmids are cryptic. Nonetheless, the small plasmids are characterized by a stable inheritance in *Synechocystis* populations; as exemplified by shuttle vectors derived from pCC5.2, which are stable in *Synechocystis* over 50 generation or 30 days without selection pressure (Jin et al., 2018; Xiao et al., 2018). Whether small plasmid stability in *Synechocystis* populations is supported by horizontal transfer remains unknown. Here, we study the mechanisms involved in horizontal transfer of plasmid DNA or whole plasmid replicons in the evolution of *Synechocystis* small cryptic plasmids.

## EXPERIMENTAL PROCEDURES

### Phylogenetic analysis

The search for putative homologous proteins of the pCA2.4, pCB2.4, and pCC5.2 ORFs was conducted by using the online version of BLAST v2.13.0 (Boratyn et al., 2013) against the non‐redundant protein sequences database (06/2022). BLAST results were filtered using thresholds of >30% sequence identity, >70% query coverage, E‐value <10^‐9^ and gene presence in plasmid genomes. A selection of representative protein sequences was aligned with MAFFT v7.487 using the default parameters (Katoh & Standley, 2013). The phylogenetic tree inference was performed using IQ‐TREE v2.1.4‐beta (Minh et al., 2020) with LG model and 1000 bootstrap replicates. Constrained phylogenies were performed for pCA2.4 using the constraint (Firmicutes, Proteobacteria, Cyanobacteria), and for pCC5.2 using the constraint (Oscillatoriales, Nostocales, remaining Cyanobacteria). Cyanobacteria species taxonomy was determined according to Strunecký et al. (2023). The likelihoods of the constrained and unconstrained tree topologies were statistically compared using the approximately unbiased (AU) test (Shimodaira, 2002) with 10,000 bootstrap replicates using IQ‐TREE. The resulting maximum‐likelihood phylogenetic tree was plotted with iTOL v6.5.8 (Letunic & Bork, 2016).

### Comparative transcriptomics

Transcriptomes were downloaded from the Sequence Read Archive (SRA; ver. 10/2020) of NCBI (Leinonen et al., 2010). The datasets were filtered to include only paired‐end fastq‐formats deriving from Illumina‐sequencing. RNA‐Seq libraries deviating from the standard RNA‐Seq approach such as miRNA‐Seq were excluded from the analysis. Quality checks were performed before and after trimming using FastQC v0.11.9 (Andrews et al., 2010) and MultiQC v1.9 (Ewels et al., 2016). The read quality was improved by applying Trimmomatic v0.39 (Bolger et al., 2014) with these parameters: SLIDINGWINDOW:4:15 MINLEN:36 LEADING:3 TRAILING:3. The transcriptomic datasets were mapped to the reference genome (GCF_000340785.1) using Bowtie2 v2.3.5.1 (Langmead & Salzberg, 2012). In order to evaluate the mapping quality, the alignment results were converted to bam format with SAMtools v1.10 (Li et al., 2009) and visualised with the Integrative Genomics Viewer v11.0.8 (Robinson et al., 2011). Twelve BioProjects having poor mapping quality were excluded (see final list in Table S1). Plasmid ORFs previously described (Xu & McFadden, 1997; Yang & McFadden, 1993, 1994) but missing in the pCA2.4, pCB2.4, and pCC5.2 annotation of the reference genome, were added manually to the annotation files. The calculation of the transcripts per million as a normalized transcription level measurement was performed with Salmon v1.5.2 (Patro et al., 2017). Statistical tests of trends in the transcription level were performed with R version 4.0.0. The package ‘PMCMRplus’ was used to perform a Skillings–Mack test in a balanced incomplete block design (Chatfield & Mander, 2009).

### Strain cultivation

*Synechocystis* was cultivated in BG11 medium at 30°C, 150 rpm, and constant light (30 μmol m^−2^ s^−1^). Culture volume was either 1 mL in a 24‐well plate or 20 mL in a 50 mL standing cell culture flask. For the selection of transformants BG11 agar plates were supplemented with 50 μg/mL kanamycin, 10 μg/mL chloramphenicol and/or 5 μg/mL each of both streptomycin and spectinomycin, depending on the respective experiment.

### *Plasmid and* Synechocystis *mutant construction*

All plasmids used in this study (Table S3) were generated using Gibson assembly and sequence integrity was verified by Sanger sequencing (Eurofins, Ebersberg, Germany). For pTB1, about 500 bp upstream and downstream homology regions of pCA2.4 were amplified using primers 1 + 2 or 5 + 6 (Table S4), respectively, and ligated into pJET1.2/blunt flanking the *aadA1* resistance cassette (amplified with primers 3 + 4). For pTB2, about 500 bp upstream and downstream homology regions of pCB2.4 were amplified using primers 7 + 8 or 9 + 10, respectively, and ligated into pJET1.2/blunt flanking the *aadA1* resistance cassette (amplified with primers 3 + 4). For pTB3, a sequence encompassing 128 bp upstream of *comEA* (*slr0197*) and the first 359 bp of the *comEA* ORF were amplified using primers 11 + 12. This sequence was then ligated into pJET1.2/blunt together with a sequence encompassing 360–844 bp of the *comEA* ORF (amplified using primers 15 + 16) and the *cat* resistance cassette (amplified using primers 13 + 14) that was flanked by both homologous sequences. For pTB4, about 800 bp upstream and 900 bp downstream homology regions of *comEC* (*sll1929*) were amplified using primers 17 + 18 or 19 + 20, respectively, and ligated into pJET1.2/blunt flanking the *cat* resistance cassette (amplified with primers 13 + 14). For pTB5, about 1000 bp upstream and 1100 bp downstream homology regions of *pilA1* (*sll1694*) were amplified using primers 21 + 22 or 23 + 24, respectively, and ligated into pJET1.2/blunt flanking the *cat* resistance cassette (amplified with primers 13 + 14). For pTB6, about 1000 bp upstream and downstream homology regions of *pilQ* (slr1277) were amplified using primers 25 + 26 or 27 + 28, respectively, and ligated into pJET1.2/blunt flanking the *cat* resistance cassette (amplified with primers 13 + 14). For pJET‐∆*recJ*‐nptI, 2000 bp upstream and downstream homology regions of *recJ* (sll1354) were amplified using primers 29 + 30 or 33 + 34, respectively, and ligated into pJET1.2/blunt flanking the *nptI* resistance cassette (amplified with primers 31 + 32).

*Synechocystis* mutants were generated by natural transformation of *Synechocystis* WT or *Synechocystis* pCA or pCB donor strains (see Table S2) according to (Xiao et al., 2018) with the respective plasmids (Table S3) and complete segregation of inserted sequences was verified by colony PCR (cPCR) of exconjugants after three of four rounds of re‐streaking on selective BG11 plates. We note that integration of antibiotic resistance marker and other genetic elements in pCA2.4 and pCB2.4 has been described earlier (Armshaw et al., 2015; Liu & Pakrasi, 2018) and for modified pCA2.4 plasmid stability without selective pressure was verified (Armshaw et al., 2015).

### PCR to test for transformation and presence of small plasmids

*Synechocystis* cell lysate was used as a template in a PCR using DreamTaq polymerase (Thermo Scientific, USA) according to manufacturers' instructions. Transformation and complete segregation of the resistance marker in all copies of plasmid or chromosome was tested by PCR using appropriate primer combinations (Table S4). Tests for the presence of small plasmids in Kiel and Sevilla labtype were performed accordingly with primer pairs amplifying the complete respective plasmid sequence (Table S4).

### DNA transfer

Donor and recipient were inoculated together in a ratio of 1:1 in 1 mL BG11 medium (24‐well plate) from a preculture cultivated until OD_750nm_ of ca. 0.2. Pre‐cultures with OD_750nm_ = 0.2 were diluted 1:1000 (cultures of slightly higher or lower OD_750nm_ were adapted accordingly by dilution). Cocultures of donor and recipient were cultivated for 7 days under standard growth conditions. Strains were grown without or with the presence of DNase I (Roche Diagnostics, Switzerland; 1 μg/mL), which was added every day during the experiment. In suspension culture, the Sevilla labtype has the tendency to form aggregates, thus co‐cultures were mixed thoroughly by pipetting once a day. Subsequently, 900 μL of the coculture were plated on BG11 plates supplemented with kanamycin (50 μg/mL), streptomycin (5 μg/mL), and spectinomycin (5 μg/mL) to quantify the number of transfer events by colony forming units (CFU). 100 μL of a 10^−5^ dilution of the remaining cell suspension was plated on BG11 plates without antibiotics to determine the total cell number by CFU.

## RESULTS

### *Evidence for the presence of cryptic plasmids in* Synechocystis *lab strains*

**FIGURE 1:**
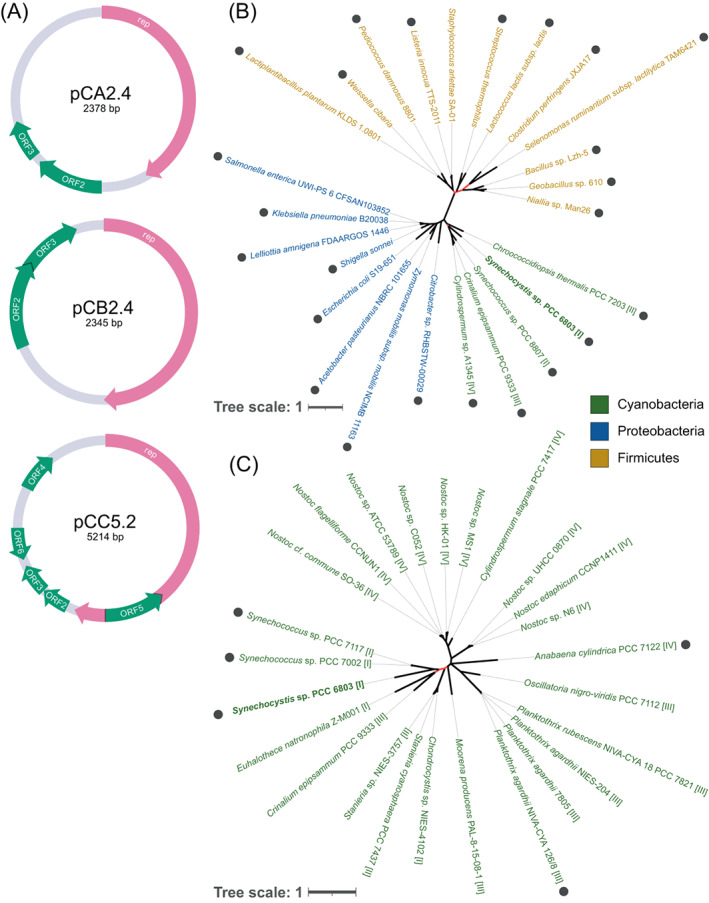
Cryptic plasmid annotation and phylogeny of the pCA2.4 and pCC5.2 replication initiation proteins. (A) Annotations of open reading frames (ORFs) in pCA2.4, pCB2.4, and pCC5.2. The annotations were generated combining previous reports in the literature (Xu & McFadden, 1997; Yang & McFadden, 1993, 1994) and the reference genome (GCA_000340785.1). Plasmid encoded Reps are identified in at least one source and are highlighted in pink. (B) Constrained phylogeny of pCA2.4 Rep using representative homologues (see full list in Supplementary File S1). (C) Constrained phylogeny of pCC5.2 Rep using representative homologues (see full list in Supplementary File S1). The operational taxonomic units (OTUs) are coloured according to the host phylum (see legend on the left). Red branches in the tree correspond to branches having a low bootstrap support (<70%). Homologues encoded in small plasmids (<25 kbp) are marked by filled circles.

A total of nine *Synechocystis* genome assemblies are reported in NCBI RefSeq and GenBank database (July 2021), of which a single assembly includes evidence for all seven plasmids reported so far in that strain (GCA_000340785.1; Trautmann et al., 2012). The small cryptic plasmids (Xu & McFadden, 1997; Yang & McFadden, 1993, 1994) encode, each, a replication initiation protein (Rep) and open reading frames (ORFs) encoding for unknown functions (Figure 1A). To gain insights into the cryptic plasmid origin, we reconstructed the plasmid‐encoded Rep phylogeny. Our search for Rep homologues yielded multiple putative homologues to the pCA2.4 Rep in plasmids residing in firmicutes, proteobacteria, and cyanobacteria; most of these plasmids are characterized by small size. The phylogenetic reconstruction of pCA2.4 Rep showed clear splits among the main phyla (Figure 1B). Furthermore, the likelihood of a phylogeny reconstructed while constraining the splits among the main phyla was not significantly different from the likelihood of the unconstrained phylogeny (*P* = 0.538; using the approximately unbiased [AU] test). The phylogenetic tree topology indicates that the evolution of pCA2.4 plasmid backbone is governed by vertical inheritance at the phyla level. The search for pCB2.4 Rep homologues detected only a few hits within *Synechocystis* substrain plasmids, thus we consider pCB2.4 plasmid backbone specific to *Synechocystis*. The search for homologues to pCC5.2 Rep yielded several putative distantly related homologues in cyanobacterial plasmids.

Those plasmids are characterized by variable sizes ranging between 4675 bp and 805,120 bp with a minority of small plasmids. A phylogenetic reconstruction of pCC5.2 Rep was performed while constraining two splits that correspond to species in the orders Nostocales and *Oscillatoriales*. The likelihood of the resulting phylogeny was not significantly different from the likelihood of the unconstrained phylogeny (*P* = 0.215; using the approximately unbiased [AU] test). The clustering of pCC5.2 Rep homologues according to the main *Cyanobacteria* orders in the phylogeny indicates that the pCC5.2 evolved mainly by vertical inheritance (Figure 1C). The search for homologues to the other cryptic plasmids' ORFs yielded no significant hits outside *Synechocystis*.

**FIGURE 2:**
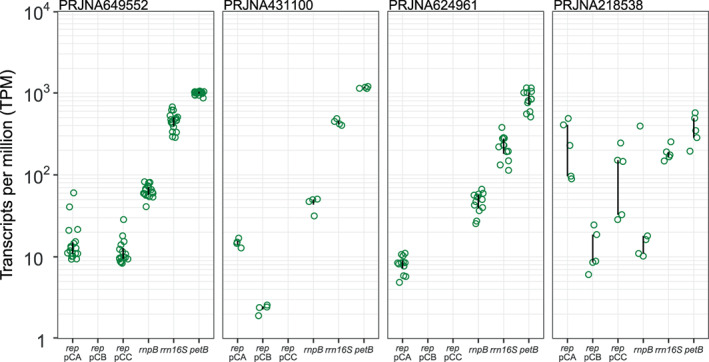
Comparison of transcription level among cryptic plasmid *rep* genes and three housekeeping genes. The underlying data corresponds to four transcriptomics studies (i.e., NCBI BioProjects) of *Synechocystis* cultured under various growth conditions in different laboratories (Bi et al., 2018; Cheng et al., 2020; García‐Cañas et al., 2021; Lau et al., 2014; see Table S1). Data points show transcript per million (TPM) per gene. Error bars show the interquartile range.

Several *Synechocystis* substrain genome assemblies contain a subset of the seven plasmids or no plasmids at all. Nonetheless, the absence of plasmid sequences in genome assemblies does not necessarily correspond to evidence for plasmid loss. For instance, pCB2.4 and pCC5.2, which were initially absent in one assembly, could be detected by PCR in the corresponding strain (Trautmann et al., 2012). Small plasmids may be absent from sequenced genomes due to technical reasons (Juraschek et al., 2021). Since all *Synechocystis* cultures are descendants of the same isolate (reported in Stanier et al., 1971), it is likely that the *Synechocystis* cryptic plasmids are more prevalent than their distribution as reflected in published *Synechocystis* genomes. To further study evidence for the small plasmids in *Synechocystis*, we investigated the transcription level of plasmid‐encoded genes using a meta‐analysis of publicly available transcriptomics datasets. Here, we focused on the transcription level of the plasmid‐encoded replication initiation protein (Rep) as evidence for the cryptic plasmid replication in the host. The *rep* transcription level was compared to the transcription level of three chromosome‐encoded reference genes: *petB* (cytochrome b6), *rrn16S* (ribosomal 16S RNA), and *rnpB* (RNA subunit of ribonuclease P; Pinto et al., 2012). Our results reveal evidence for pCA2.4 presence in all inspected datasets, while evidence for pCB2.4 and pCC5.2 was found in only two of the datasets (Figure 2). An examination of the plasmid‐encoded gene transcription level revealed several combinations of the small cryptic plasmids in *Synechocystis* cultures across the four datasets: (1) pCA2.4 and pCC5.2 (PRJNA649552), (2) pCA2.4 and pCB2.4 (PRJNA431100), (3) only pCA2.4 (PRJNA624961), or (4) all three cryptic plasmids (PRJNA218538). Evidence for the cryptic plasmid *rep* transcription was robust among replicates within the same dataset, hence the absence of evidence for the cryptic plasmids is likely due to plasmid loss prior to the experiment (rather than during the experiment). The *rep* transcription level was significantly different among the three small cryptic plasmids (*P* = 7.552e‐05, using Skillings–Mack test) in the four BioProjects, with pCB2.4 having the lowest *rep* transcription level. Furthermore, the median transcription level of the cryptic plasmids' *rep* was lower than that of the three compared reference genes. The evidence for robust transcription of the cryptic plasmid *rep* in *Synechocystis* cultures supports the notion that cryptic plasmid maintenance is not always well documented in *Synechocystis* sequenced genomes.

### *Loss and persistence of small cryptic plasmids in* Synechocystis *substrains*

To further validate the cryptic plasmid loss and the persistence of different plasmid combinations, we examined cultures having two of the plasmid combinations observed in the comparative transcriptomics. Testing the plasmid content in *Synechocystis* cultures used in BioProject PRJNA649552 (García‐Cañas et al., 2021) by PCR confirmed the absence of pCB2.4 in those cultures (Figure S1A), which we term here the ‘Sevilla labtype’ (the relevant strain originated from the lab of Francisco Javier Florencio Bellido in Sevilla, Spain). Furthermore, we found that our local *Synechocystis* culture, termed here ‘Kiel labtype’, harbours only pCA2.4 and pCB2.4 (similarly to BioProject PRJNA431100 in Figures 2, S1A).

To examine whether a cryptic plasmid can persist in a *Synechocystis* labtype that lost that plasmid, we tested the stability of pCB2.4 maintenance in the Sevilla labtype. For that purpose, we generated pCB2.4ΩSmR—that is pCB2.4 from the Kiel labtype with an insertion of an antibiotic resistance marker *aadA1*, which lends resistance against streptomycin (*SmR*) and spectinomycin (hereafter termed pCB). The Sevilla labtype was transformed with pCB and successful plasmid uptake was tested by antibiotic resistance of the selected transformants and PCR amplification of the complete recombinant plasmid (Figure S1B). Quantifying the stability of plasmid pCB in non‐selective media over 8 days showed that the plasmid was maintained in ca. 90% of the hosts in both Sevilla and the Kiel labtypes (Figure S2). Furthermore, the growth dynamics of both Kiel and Sevilla labtypes with or without the recombinant pCB were comparable over the duration of 8 days. Our results thus show that the cryptic plasmid pCB is able to persist in the Sevilla as well as in the Kiel labtype, albeit its stability is impaired.

### *Gene transfer among cryptic plasmids within* Synechocystis *populations is mediated by natural transformation*

To further study the mechanisms involved in the maintenance of cryptic plasmids we investigated the potential of those plasmids to transfer between cells. The small *Synechocystis* plasmids are lacking genes that encode for mobility mechanisms, hence they are unlikely to be transferred by conjugation. Furthermore, both Kiel and Sevilla labtypes do not harbour phages hence plasmid transfer via transduction (e.g., as reported in *Staphylococcus*; Humphrey et al., 2021) can be ruled out. Since *Synechocystis* is naturally competent (Grigorieva & Shestakov, 1982), plasmid DNA is likely to be transferred by natural transformation. Additionally, *Synechocystis* is known to produce OMVs (Pardo et al., 2015), which were suggested to contribute to plasmid transfer in other bacteria (e.g., Tran & Boedicker, 2019).

**FIGURE 3:**
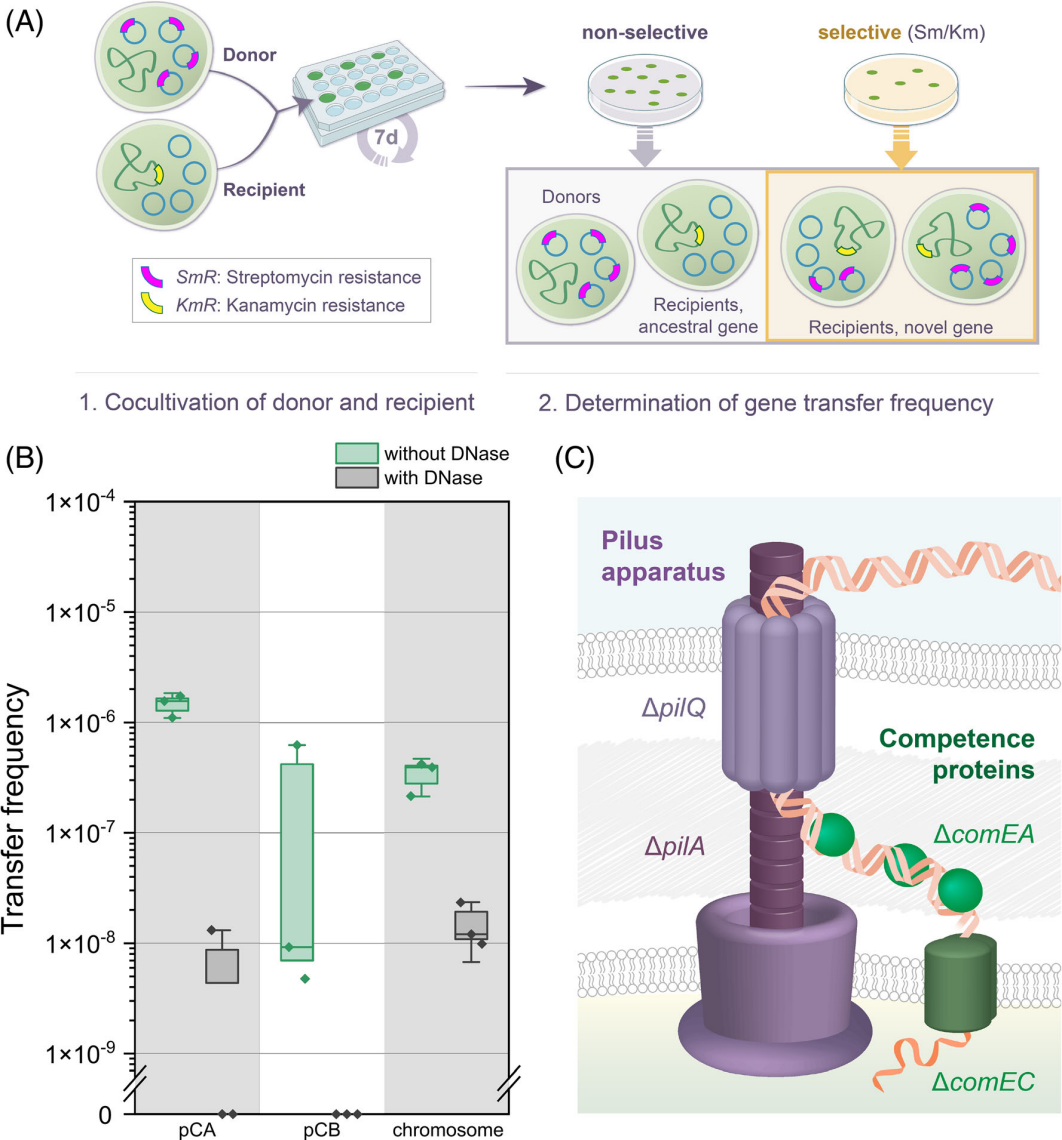
Gene transfer in *Synechocystis* cultures via natural transformation. (A) An illustration of our experimental setup for testing and quantifying plasmid‐encoded gene transfer in *Synechocystis*. The donor and recipient were co‐cultivated for 7 days. The donor was deficient in natural competence; thus, the transfer was unidirectional from donor to recipient. After 7 days the co‐culture was plated on non‐selective media for cell number determination. The marker gene transfer into the recipient was quantified by plating on double selective plates. (B) Transfer frequency of pCA or pCB encoded *SmR* marker, or a chromosomal encoded *CmR* marker from donor to the recipient WT* with or without DNase treatment (*n* = 3). Transfer frequency is calculated by the number of transformed recipient cells (double resistance to streptomycin/spectinomycin or chloramphenicol and kanamycin) divided by total cell number. Boxes indicate one standard error; whiskers indicate two standard errors. (C) An illustration of the DNA uptake machinery in *Synechocystis*. Type IV pilus (purple) and competence proteins (green) are essential for DNA transport across the outer and inner cell membranes. Knockout mutants of *pilA1* (major pilus subunit), *pilQ* (outer membrane pore), *comEA* (DNA binding in periplasm), and *comEC* (inner membrane transport) as recipient cells are deficient in DNA uptake in marker gene transfer experiments.

To test for the occurrence of plasmid DNA transfer, we established an experimental system where the transfer frequency of pCA2.4 and pCB2.4 encoded genes between donors and recipients can be followed and quantified (Figure 3A). For that purpose, we generated another plasmid: pCA2.4ΩSmR, by cloning the antibiotic resistance marker *aadA1* into pCA2.4 from the Kiel labtype (termed hereafter pCA). The two plasmid donors in our experimental setup carry either pCA or pCB. The recipient in our setup encodes a kanamycin resistance gene (*KmR*), which was introduced into a neutral locus (Kunert et al., 2000; termed here WT*; See Table S2 for details on donors and recipients used in this study). Using our newly established system, DNA transfer events were consequently quantified by the frequency of transformed recipients; those are cells that are resistant to both streptomycin/spectinomycin and kanamycin. Donors and recipients of the Kiel labtype were inoculated together in a new culture and then cultivated for 7 days during their exponential growth phase. The transfer frequency of the *SmR* resistance gene was estimated from the proportion of transformed recipients based on colony counts of the cultured populations plated on double‐selective plates (kanamycin and streptomycin/spectinomycin; Figure 3A). In order to enable only uni‐directional transfer of the marker genes from donors to recipients, the donors were rendered natural competence deficient via a *comEA* knockout (Yoshihara et al., 2001) through insertion of a chloramphenicol resistance gene (*CmR*) into the *comEA* ORF. In addition, the integration of *CmR* in the donor's chromosome enabled us to compare the transfer of the chromosome *CmR* encoded gene in the same experimental approach as the transfer of the small plasmid‐encoded *SmR* gene (by selecting for colonies double resistant towards chloramphenicol and kanamycin).

Our results reveal that plasmid‐ and chromosome‐encoded genes are horizontally transferred in the Kiel labtype. The pCA‐mediated transfer frequency ranged between 1 × 10^−6^ and 2 × 10^−6^ and showed a small variability among replicates (Figure 3B). In comparison, the pCB transfer frequency was lower and more variable (ranging between 4 × 10^−9^ and 7 × 10^−7^). The transfer frequency of the chromosome‐encoded marker ranged between 2 × 10^−7^ and 5 × 10^−7^, and is not significantly different from the pCA and pCB transfer frequency (*P* = 0.061, using Kruskal–Wallis test). Thus, in the absence of plasmid mobility mechanisms, the frequency of gene transfer via natural transformation is similar between plasmid‐ and chromosome‐encoded genes.

To gain further insight into the horizontal gene transfer mechanism, we examined the contribution of freely available DNA in the environment to the horizontal transfer frequency. For that purpose, we added DNase during the donors and recipients' co‐cultivation period. Our results show that the addition of DNase reduces the transfer frequency of the pCA‐ and chromosome‐encoded antibiotic resistance gene by one to two orders of magnitude and abolishes the transfer of the pCB‐encoded antibiotic resistance gene (Figure 3B). The effect of DNase treatment on the transfer frequency implies that the presence of unprotected DNA in the culture media is essential for the gene transfer observed in our experiment, as one would expect for DNA transfer via natural transformation. To further validate natural transformation as the transfer mechanism at play, we constructed knockout mutants for various components of the natural competence machinery including the genes *pilA1*, *pilQ*, *comEA*, and *comEC* (Figure 3C). According to the currently accepted model for the natural competence machinery in gram‐negative bacteria, the generated knockout mutants are deficient either in DNA transport across the outer membrane (pilus mutants) or the inner membrane (competence protein mutants; Figure 3C; reviewed in Dubnau & Blokesch, 2019). Previous studies of *Synechocystis* additionally showed that the *pilA1*, *pilQ*, and *comEA* knockout mutants are deficient in DNA uptake via natural transformation (Yoshihara et al., 2001). Repeating the transfer experiment with the competence‐knockout mutants as recipients showed that plasmid‐ and chromosome‐encoded gene transfer was abolished in all tested mutants. Our results demonstrate that natural competence is essential for the gene transfer observed in our experiment, and the horizontal DNA transfer in the *Synechocystis* populations is mediated solely by natural transformation.

### Plasmid gene transfer frequency depends on the recipient labtype and is higher for pCA compared to pCB

The finding of a *Synechocystis* labtype (Sevilla), which is lacking pCB2.4, enabled us to further examine the transfer frequency of plasmid‐encoded genes and whole plasmid replicons under the same conditions. For that purpose, we performed additional transfer experiments with the Kiel and Sevilla labtype as recipients for pCA and pCB. Note that in these experiments, transfer of the pCB‐encoded antibiotic resistance gene into the Sevilla labtype corresponded to plasmid transfer, while the transfer into the Kiel labtype corresponded to either plasmid or gene transfer.

**FIGURE 4:**
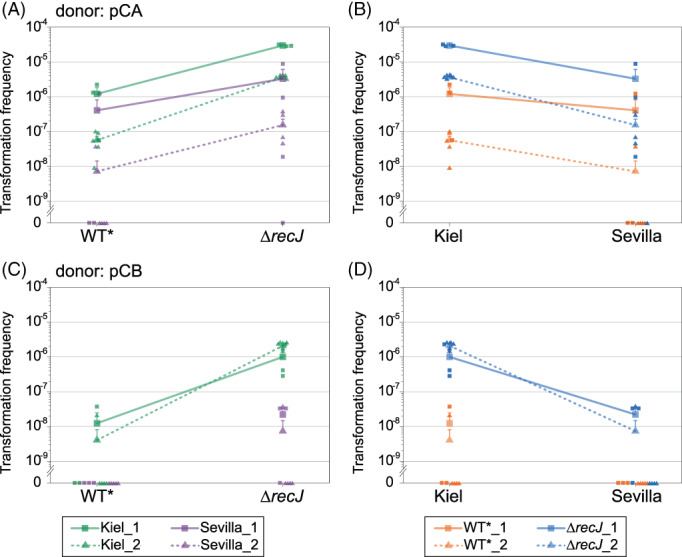
Transfer frequency of pCA and pCB encoded antibiotic resistance genes from donor to recipient strains. Transfer frequency is shown according to recipient genotype (left: WT* or ∆*recJ*) or labtype (right: Kiel or Sevilla) and the donor plasmid (top: pCA; bottom: pCB). The Kiel WT* is the same recipient as in Figure 3B. Transfer frequency is calculated by the number of transformed recipient cells (double resistant to streptomycin/spectinomycin and kanamycin) divided by total cell number. The results of two independent experiments (solid lines and squares; dashed lines and triangles) are presented, with three or five technical replicates, respectively.

In order to improve our ability to observe rare transformation events, we constructed an additional recipient genotype where the *recJ* locus was replaced by a *KmR* cassette (termed *∆recJ*). Previous studies reported that the knockout of the *recJ* gene, which encodes for a single‐strand exonuclease, increased transformation rate into *Synechocystis* by two orders of magnitude (Kufryk et al., 2002). Our setup thus included four recipient types that were the combination of the two *Synechocystis* labtypes, Kiel and Sevilla, and the two genotypes, WT* and *∆recJ*. Transformation experiments were performed with all four recipient types and marked plasmids (Figure 4).

Our results show that transformation frequency of the pCA‐encoded antibiotic resistance gene was variable between the two independent experiments, while the effect of both genotype and labtype had the same tendency. The transfer frequency in the *∆recJ* recipient was higher in comparison to the WT* recipient in both Kiel and Sevilla labtypes. The increased transformation frequency in *∆recJ* genotype adds support to their utility for the documentation of rare transformation events. Furthermore, the pCA‐encoded gene transfer frequency into the Sevilla labtype was, on average, lower in comparison to the Kiel labtype regardless of the genotype (Figure 4A, B).

The transfer frequency of the pCB‐encoded antibiotic resistance gene had a small variation between the experiments and was overall lower compared to pCA, confirming our previous results (Figure 3B). Additionally, the pCB transfer frequency into the Kiel labtype recipients was elevated in the *∆recJ* genotype compared to the WT* genotype (Figure 4C, D). In the Sevilla labtype, we detected no pCB transfer into the WT* genotype, while transfer into the *∆recJ* recipient could be detected. The observation of pCB transfer in the Sevilla *∆recJ* genotype demonstrates that the complete pCB plasmid can be transferred via natural transformation in the *Synechocystis* population, albeit at a low frequency. The low pCB transfer frequency may be due to the generally lower DNA uptake frequency in the Sevilla labtype or due to differences between gene transfer and plasmid reconstitution.

Comparing the results of the transformation frequency between the two plasmids reveals several differences: the high variability between experiments observed for pCA suggests that transfer of pCA is more sensitive to environmental factors (i.e., culture conditions) in comparison to pCB. The overall lower transformation frequency observed for pCB is unlikely to be related only to differences in the plasmid copy number, since pCA2.4 copy number in *Synechocystis* is approximately two‐fold higher than that of pCB2.4 (Berla & Pakrasi, 2012), while the difference in transformation frequency is higher than that. The lower transformation frequency observed for the Sevilla recipient, independent of the plasmid, could be explained by different competence dynamics between the two labtypes. Taken together, our results show that plasmid DNA transfer occurs frequently inside a *Synechocystis* population via natural transformation.

## DISCUSSION

**FIGURE 5:**
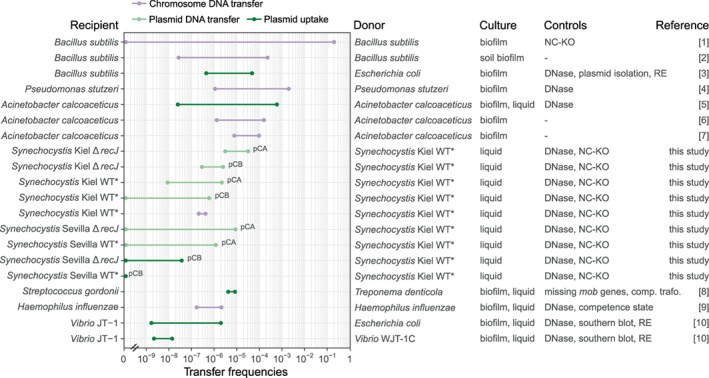
Transfer frequencies for plasmid uptake and horizontal transfer of plasmid and chromosome encoded traits during cocultivation of the same or different species. Transfer frequencies from this study and literature are ordered by the recipient from high to low on a logarithmic scale. Sensitivity to DNase is the most common control (conjugation or transduction are not sensitive to DNase added to the media). For transfer of chromosomal markers in several studies the DNase treatment was not successful or not applied. For the estimation of plasmid‐mediated transfer frequency, controls to exclude other routes of horizontal transfer are essential, which are not necessarily required for chromosome‐mediated transfer (previous studies on plasmids missing these controls were not included). Additional controls are functional knockouts of the natural competence machinery (NC‐KO), plasmid isolation from the recipient, digestion of restriction enzymes (RE), missing *mob* genes on the transferred plasmid, comparison of transformation characteristics with naked plasmid, experiments in dependency of the competence state, southern blot. Species‐specific properties in natural competence and differences in experimental settings (e.g., transformation protocol, incorporated DNA, cultivation in biofilms or suspension cultures) should be taken into account during the comparison. Studies in which transfer was not quantified as a ratio of living cells were not included in this summary (e.g., studies with transfer frequencies given as ratio of transformed cells to culture volume). We note that the combination of the generally lower transfer frequencies for both plasmid pCB and Sevilla labtype could lead to an underestimation of the general plasmid uptake ability of *Synechocystis*. References: 1. (Zhang et al., 2018), 2. (Graham & Istock, 1978), 3. (Wang et al., 2007), 4. (Stewart et al., 1983), 5. (Rochelle et al., 1988), 6. (Vakeria et al., 1985), 7. (Williams et al., 1996), 8. (Wang et al., 2002), 9. (Albritton et al., 1982), 10. (Paul et al., 1992).

The transfer of chromosome‐encoded genes via natural transformation within bacterial populations has been extensively studied in naturally competent organisms. In our study, we compare the transfer frequency among replicons and labtypes. A comparison of our results to previous measurements of transfer frequencies shows that the frequencies we report here are modest in comparison to the high transfer frequencies described for *Bacillus subtilis* or *Pseudomonas stutzeri* (Figure 5). We note, however, that most of the higher previously reported transfer frequencies were measured in biofilm assays, while our measurements were performed with *Synechocystis* cultivated in suspension culture. The comparison to other studies further shows that the recipient identity is a main determinant of transfer frequency via natural transformation. Differences among species, for example, *B. subtilis* and *Synechocystis*, may be explained by various factors that affect the frequency of competent recipient cells, DNA uptake, and integration (reviewed in Dubnau & Blokesch, 2019; Huang et al., 2021; Johnston et al., 2014), as well as the experimental setup (e.g., measurements performed in biofilms versus suspension cultures). The difference we observed between the Kiel and Sevilla labtypes shows that such differences in transformation frequency exist also among closely related *Synechocystis* labtypes. The frequency we report here for *Synechocystis* chromosome‐ and plasmid‐encoded gene transfer in the two *Synechocystis* labtypes (Figures 3, 4) are within the range of previously published intermediate frequencies. Our study further reveals that the transfer of chromosome‐ and plasmid‐encoded genes occurs with similar frequencies in *Synechocystis*. Our study thus suggests that the frequency of DNA transfer via natural transformation is not dependent on the replicon type (i.e., plasmid of chromosome).

Acquisition of new plasmids via natural transformation is generally possible as our results and previous studies show. In our experiments, the transfer of a pCB‐encoded gene via plasmid uptake was documented only in the Sevilla *∆recJ* recipient in very low frequencies. Using our data, it is however difficult to conclude if those lower plasmid uptake frequencies were solely due to inherent difficulties in plasmid reconstitution or an effect of the labtype. In other species, the frequency of plasmid uptake is in the range of chromosome‐encoded genes (e.g., as for in *B. subtilis* and *A. calcoaceticus*, Figure 5; Graham & Istock, 1978; Rochelle et al., 1988; Vakeria et al., 1985; Wang et al., 2007; Williams et al., 1996; Zhang et al., 2018). The low frequency of plasmid uptake observed in our experiments is comparable to plasmid uptake frequencies reported in *Vibrio* (Paul et al., 1992). A successful plasmid reconstitution following transformation depends on the availability of complementary strands for recombination, which may be absent during the acquisition of a new plasmid. Indeed, several mechanistic differences between pathways for chromosomal DNA and whole plasmid acquisition have been described (e.g., Serrano et al., 2018). Barriers for whole plasmid transfer may be related to inherent difficulties of plasmid reconstitution following natural transformation (reviewed in Lorenz & Wackernagel, 1994), as well as the presence of defence mechanisms against incoming plasmids (e.g., recently described plasmid defence systems in the naturally competent and pathogenic strains of *Vibrio cholerae*; Jaskólska et al., 2022).

Plasmid persistence in the population over time depends on several factors, including the stability of plasmid inheritance, plasmid mobility, population structure, and the selection regimes (Wein & Dagan, 2020). Considering the frequency of plasmid uptake via natural transformation (Figures 4, 5), we posit that cryptic plasmid persistence in *Synechocystis* is unlikely to be supported by natural transformation. Alternative strategies for the persistence of non‐mobile (or non‐mobilizable) cryptic plasmids are, for instance, the evolution of stable plasmid inheritance within the host or the presence of plasmid addiction mechanisms (reviewed in Wein & Dagan, 2020). Nonetheless, plasmid genome evolution in naturally competent organisms likely includes frequent genetic recombination within the population that has the potential to either increase the frequency of selectively advantageous alleles—or more often—eliminate genetic variation within the population.

## AUTHOR CONTRIBUTIONS

**Fabian Nies:** Conceptualization (equal); investigation (lead); methodology (lead); project administration (lead); validation (lead); visualization (lead); writing – original draft (equal). **Tanita Wein:** Conceptualization (equal); investigation (equal); methodology (equal); validation (equal); writing – review and editing (supporting). **Dustin M. Hanke:** Conceptualization (equal); data curation (supporting); investigation (supporting); visualization (equal); writing – review and editing (supporting). **Benjamin L. Springstein:** Conceptualization (equal); investigation (supporting); methodology (supporting); writing – review and editing (supporting). **Jaime Alcorta:** Formal analysis (supporting); investigation (supporting); writing – review and editing (supporting). **Claudia Taubenheim:** Investigation (supporting); writing – review and editing (supporting). **Tal Dagan:** Conceptualization (equal); funding acquisition (lead); project administration (equal); supervision (lead); visualization (supporting); writing – original draft (equal).

## CONFLICT OF INTEREST STATEMENT

The authors declare no conflict of interest.

## Supporting information


**Data S1:** Supporting Information

## Data Availability

All relevant data is supplied in the supplamentary material.
