# Supplementary material for: Role of natural transformation in the evolution of small cryptic plasmids in Synechocystis sp. PCC 6803
Source: Environ Microbiol Rep. 2023 Oct 4;15(6):656–68. doi: 10.1111/1758-2229.13203 (PMC10667661; doi:10.1111/1758-2229.13203)
Supplement: Supplementary file 1 — Data S1: Supporting Information [file EMI4-15-656-s001.pdf]

Role of natural transformation in the evolution of small cryptic plasmids in *Synechocystis* sp. PCC 6803

Fabian Nies, Tanita Wein, Dustin M. Hanke, Benjamin L. Springstein, Jaime Alcorta, Claudia Taubenheim, Tal Dagan

Supplemental material

Figure S1. Plasmid content of *Synechocystis* labtypes and pCB uptake into Sevilla labtype via transformation. 2

Figure S2. Stability of plasmid pCB in *Synechocystis* Kiel and Sevilla labtypes over time...... 3

Table S1. Experimental conditions of BioProjects analysed. 4

Table S2. *Synechocystis* mutants used in this study. 6

Table S3. Plasmids used in the study. 7

Table S4. Primers used in the study. 8

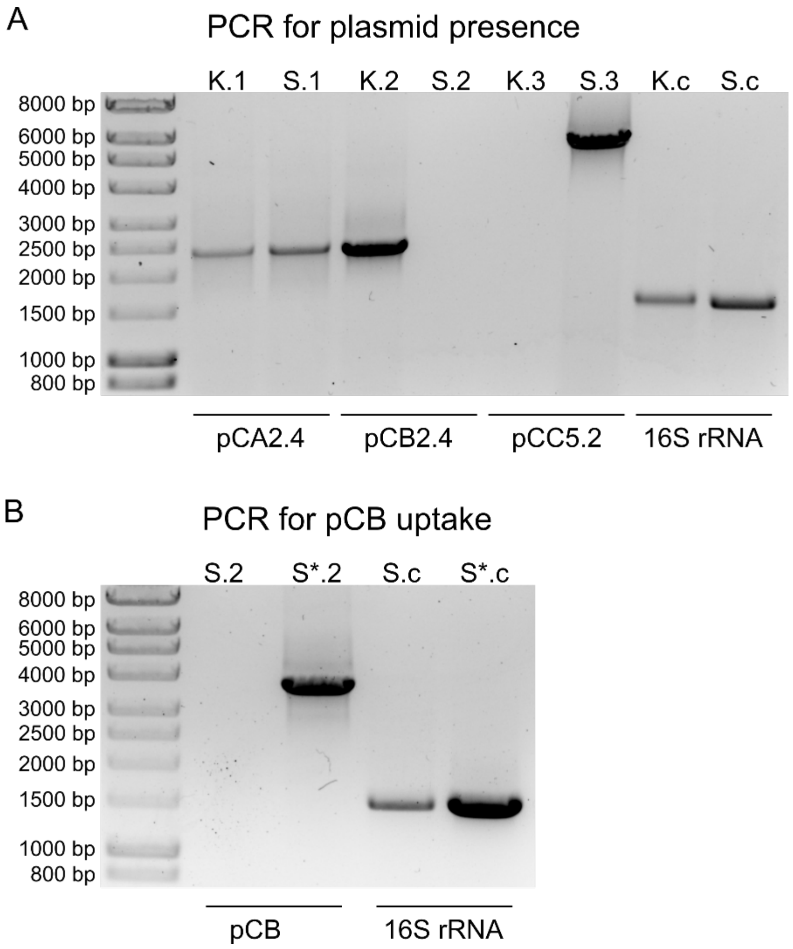

Figure S1. Plasmid content of *Synechocystis* labtypes and pCB uptake into Sevilla labtype via transformation.

A) PCR to test for small plasmid presence in *Synechocystis* Kiel (K) and Sevilla (S) labtype . pCA2.4 is present in both labtypes, pCB2.4 is only present in Kiel labtype, pCC5.2 is only present in Sevilla labtype. B) PCR to validate uptake of pCB via transformation into Sevilla labtype (S*). While pCB2.4 is absent in Sevilla labtype WT the recombinant plasmid pCB is present in the mutant S* after transformation. Additionally, Sanger sequencing of the PCR products revealed 100% identity with the original plasmid, indicating that pCB was taken up and reconstituted into the whole plasmid in S*. Primer pair 1 (1-f, 1-r) for pCA2.4 detection (expected size: 2372 bp), 2 (2-f, 2-r) for pCB2.4 and pCB detection (expected size - S: 2345 bp; S*: 3577 bp), 3 (3-f, 3-r) for pCC5.2 detection (expected size: 5208 bp), and c (27F1, 1494Rc; (Neilan et al., 1997)) for 16S rRNA gene control (expected size: 1453 bp).

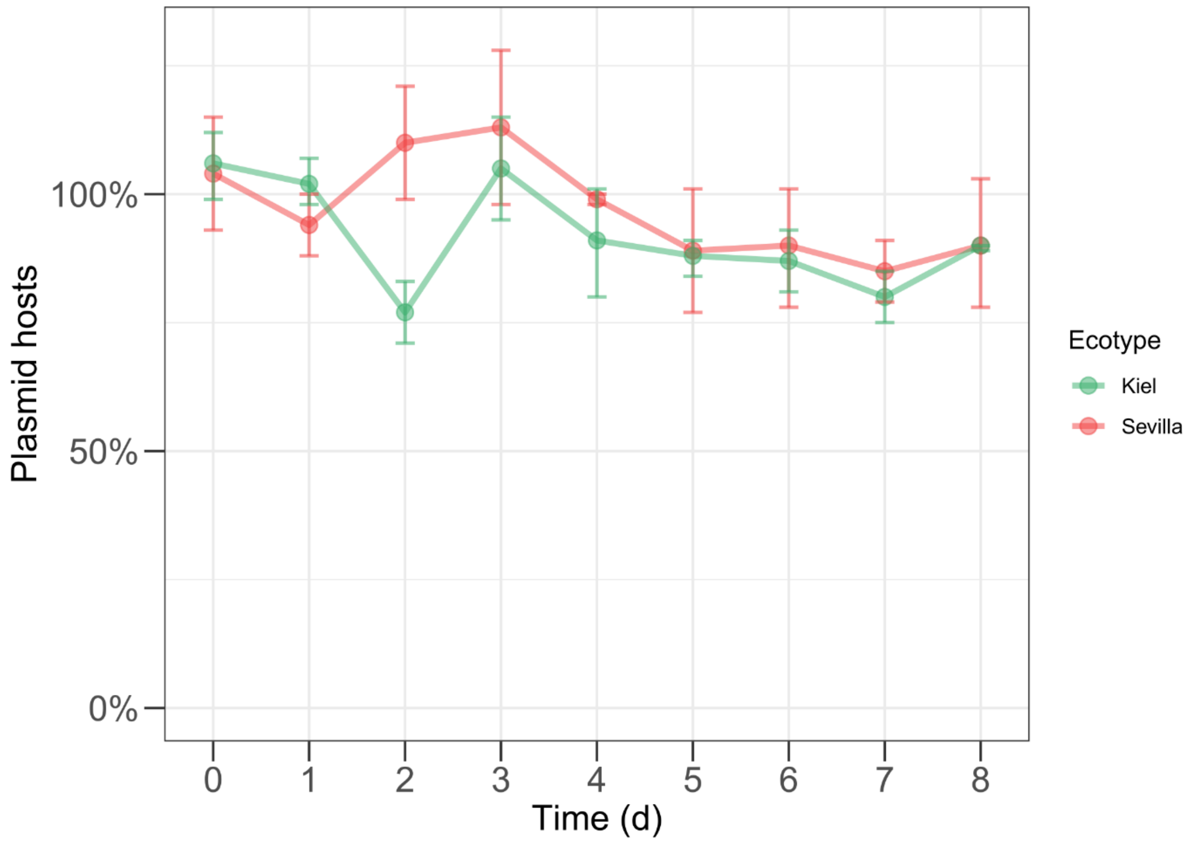

Figure S2. Stability of plasmid pCB in *Synechocystis* Kiel and Sevilla labtypes over time. Cultures of *Synechocystis* Kiel and Sevilla labtypes, each harbouring recombinant plasmid pCB, were cultivated for 8 d without selective pressure. The stability of pCB is comparable for both labtypes during the observed time. Each day 150 µl samples were collected from each culture and plated in appropriate dilutions on BG11 plates with and without antibiotics. The ratio of colonies on plates with antibiotics (pCB carrier with resistance marker) to colonies on plates without antibiotics (total population) gives the proportion of antibiotic resistance-providing plasmid hosts inside a population. The mean colony count from three biological replicates is shown together with the standard error.

Table S1. Experimental conditions of BioProjects analysed.

PRJNA218538: (Lau et al., 2014); PRJNA431100: (Bi et al., 2018); PRJNA624961: (Cheng et al., 2020); PRJNA649552: (García-Cañas et al., 2021).

BioProject	Biosample	Phenotype/Genotype	Treatment
PRJNA218538	SAMN02351218	low PHA-producing potential	
PRJNA218538	SAMN02351221	medium PHA-producing potential	
PRJNA218538	SAMN02351216	medium PHA-producing potential	
PRJNA218538	SAMN02351219	high PHA-producing potential	
PRJNA218538	SAMN02351217	high PHA-producing potential	
PRJNA649552	SAMN15668935	WT	
PRJNA649552	SAMN15668934	WT	
PRJNA649552	SAMN15668933	petR-	grown on BC11C-Cu
PRJNA649552	SAMN15668932	petR-	grown on BG11C-Cu after copper addition
PRJNA649552	SAMN15668931	petP-	grown on BC11C-Cu
PRJNA649552	SAMN15668930	petP-	grown on BG11C-Cu after copper addition
PRJNA649552	SAMN15668929	WT	grown on BC11C-Cu
PRJNA649552	SAMN15668928	WT	grown on BG11C-Cu after copper addition
PRJNA649552	SAMN15668927	petR-	grown on BC11C-Cu
PRJNA649552	SAMN15668926	petR-	grown on BG11C-Cu after copper addition
PRJNA649552	SAMN15668925	petP-	grown on BC11C-Cu
PRJNA649552	SAMN15668924	petP-	grown on BG11C-Cu after copper addition
PRJNA649552	SAMN15668923	WT	grown on BC11C-Cu
PRJNA649552	SAMN15668922	WT	grown on BG11C-Cu after copper addition
PRJNA649552	SAMN15668921	petR-	grown on BC11C-Cu
PRJNA649552	SAMN15668920	petR-	grown on BG11C-Cu after copper addition
PRJNA649552	SAMN15668919	petP-	grown on BC11C-Cu
PRJNA649552	SAMN15668918	petP-	grown on BG11C-Cu after copper addition
PRJNA624961	SAMN14589725	isiA mutant	suppressor mutant *Synechocystis* sp. PCC 6803 isiA mutant grown in Iron depleted 24h, then followed MV treated 4h

BioProject	Biosample	Phenotype/Genotype	Treatment
PRJNA624961	SAMN14589719	isiA mutant	suppressor mutant *Synechocystis* sp. PCC 6803 isiA mutant grown in Iron depleted 24h
PRJNA624961	SAMN14589718	isiA mutant	suppressor mutant *Synechocystis* sp. PCC 6803 isiA mutant grown in Iron depleted 24h
PRJNA624961	SAMN14589717	isiA mutant	suppressor mutant *Synechocystis* sp. PCC 6803 isiA mutant grown in Iron depleted 24h
PRJNA624961	SAMN14589715	WT	*Synechocystis* sp. PCC 6803 grown in Iron depleted 24h, then followed MV treated 4h
PRJNA624961	SAMN14589714	WT	*Synechocystis* sp. PCC 6803 grown in Iron depleted 24h, then followed MV treated 4h
PRJNA624961	SAMN14589713	WT	*Synechocystis* sp. PCC 6803 grown in Iron depleted 24h, then followed MV treated 4h
PRJNA624961	SAMN14589712	WT	*Synechocystis* sp. PCC 6803 grown in Iron depleted 24h
PRJNA624961	SAMN14589727	isiA mutant	suppressor mutant *Synechocystis* sp. PCC 6803 isiA mutant grown in Iron depleted 24h, then followed MV treated 4h
PRJNA624961	SAMN14589726	isiA mutant	suppressor mutant *Synechocystis* sp. PCC 6803 isiA mutant grown in Iron depleted 24h, then followed MV treated 4h
PRJNA624961	SAMN14589711	WT	*Synechocystis* sp. PCC 6803 grown in Iron depleted 24h
PRJNA624961	SAMN14589710	WT	*Synechocystis* sp. PCC 6803 grown in Iron depleted 24h
PRJNA431100	SAMN08384154	WT	
PRJNA431100	SAMN08384153	WT	
PRJNA431100	SAMN08384156	s117	
PRJNA431100	SAMN08384155	s117	

Table S2. *Synechocystis* mutants used in this study.

Donors were first transformed with the respective plasmid, which introduces the *SmR* cassette into the respective small plasmid (pCA2.4 or pCB2.4). Afterwards a *comEA* knockout was introduced to allow transfer of small plasmid encoded *SmR* marker from the donor to the recipient but not the transfer of chromosome-encoded *KmR* marker of the recipient to the donor strains. *KmR*: kanamycin resistance marker; *CmR*: chloramphenicol resistance marker; *SmR*: streptomycin/spectinomycin resistance marker.

**Labtype**	**Genotype**	**Resistance marker**	**Plasmid**	**Function**
Kiel	WT*	*KmR* (*nptI*)	pIGA	recipient
Sevilla	WT*	*KmR* (*nptI*)	pIGA	recipient
Kiel	Δ*recJ*	*KmR* (*nptI*)	pJET-Δ*recJ*-*nptI*	recipient
Sevilla	Δ*recJ*	*KmR* (*nptI*)	pJET-Δ*recJ*-*nptI*	recipient
Kiel	Δ*pilA1*	*CmR* (*cat A1*)	pTB5	competence mutant
Kiel	Δ*pilQ*	*CmR* (*cat A1*)	pTB6	competence mutant
Kiel	Δ*comEA*	*CmR* (*cat A1*)	pTB3	competence mutant
Kiel	Δ*comEC*	*CmR* (*cat A1*)	pTB4	competence mutant
Kiel	pCA<br>Δ*comEA*	*SmR* (*aadA1*)<br>*CmR* (*cat A1*)	pTB1 and pTB3	donor
Kiel	pCB<br>Δ*comEA*	*SmR* (*aadA1*)<br>*CmR* (*cat A1*)	pTB2 and pTB3	donor

Table S3. Plasmids used in the study.

Plasmids	Description	Resistance	Reference
pJET1.2/blunt	*E. coli* subcloning vector	Ampicillin (*AmpR*)	Thermo Fischer Scientific
pIGA	Cyanobacterial vector for insertion of *nptI* into neutral locus (RS1 and RS2) of *slr0168* in *Synechocystis*	*AmpR*, *KmR*	(Kunert et al., 2000)
pTB1	pJET1.2/blunt with homology flanks for insertion *aadA1* gene between the predicted *rep* and *orf2* of pCA2.4_M	*AmpR*, *SmR*, *SpR*	This study
pTB2	pJET1.2/blunt with homology flanks for insertion of the *aadA1* gene between the predicted *rep* and *orf2* of pCB2.4_M	*AmpR*, *SmR*, *SpR*	This study
pTB3	pJET1.2/blunt with homology flanks for interruption of *slr0197* ORF with the *cat* gene	*AmpR*, *CmR*	This study
pTB4	pJET1.2/blunt with homology flanks for replacement of *sll1929* with the *cat* gene	*AmpR*, *CmR*	This study
pTB5	pJET1.2/blunt with homology flanks for replacement of *sll1694* with the *cat* gene	*AmpR*, *CmR*	This study
pTB6	pJET1.2/blunt with homology flanks for replacement of *slr1277* with the *cat* gene	*AmpR*, *CmR*	This study
pJET-Δ*recJ*-*nptI*	pJET1.2/blunt with homology flanks for replacement of *sll1354* with the *nptI* gene	*AmpR*, *KmR*	This study

Table S4. Primers used in the study.

#	Given name	Sequence 5' -> 3'	Purpose
1	pASm_up_for	GATGGCTCGAGTTTTTCAGCAAGATCCATTGTTTATTAATGGTTAAAGCC	Upstream homology for pTB1
2	pASm_up_rev	TACGGCAAGGTGCTGTGCACGGATCTGAGGCGCAATCAGGGGGTTC	
3	CS.3_Fwd	GATCCGTGCACAGCACCTTG	*aadA1* gene
4	CS.3_Rev	TTATTTGCCGACTACCTTGGTGATCT	
5	pASm_down_for	GATCACCAAGGTAGTCGGCAAATAAGGTCGTTGTACGTTTCCCGT	Downstream homology for pTB1
6	pASm_down_rev	GTAGGAGATCTTCTAGAAAGATCCACTTTATCCTTAATTGGAATGGGGAC	
7	pB_1F	TCGAGTTTTTCAGCAAGATATAGAAGTACAGGTAAGCGGGC	Upstream homology for pTB2
8	pB_1R	CAAGGTGCTGTGCACGGATCCTTCGTGCCCTTGACAACC	
9	pB_2F	CCAAGGTAGTCGGCAAATAATGGTGAACGAAGTGAACGC	Downstream homology for pTB2
10	pB_2R	TGTAGGAGATCTTCTAGAAAGATGCTTGATAAGCTTTATCATAAGCGTC	
11	slr0197KO_up_A	ATTGTAGGAGATCTTCTAGAAAGATGCGAACAGGTTGCCCTGA	Upstream homology for pTB3
12	slr0197KO_up_B	AACCTCTTACGTGCCGATCACCGGTCTTTTGTTTCTCGACC	
13	CAT_A	TGATCGGCACGTAAGAGGTTC	*cat* gene
14	CAT_B	TTACGCCCCGCCCTGCCA	
15	slr0197KO_down_A	GTGGCAGGGCGGGGCGTAAGGTCCGGGTCAGATTGGT	Downstream homology for pTB3
16	slr0197KO_down_B	CTCGAGTTTTTCAGCAAGATGCACTTTAGCTGGTCTGCTG	
17	ComECKO_up_A	ATTGTAGGAGATCTTCTAGAAAGATGGGCTCCATTTGCAGTTTGC	Upstream homology for pTB4
18	comECKO_up_B2	AACCTCTTACGTGCCGATCAGCAGTTTAACTCCATATCAAGTTTCCGAAAATTTC	

#	Given name	Sequence 5’ -> 3’	Purpose
19	comECKO_down_A2	AGTGGCAGGGCGGGGCGTAAACTATAGTCATTTCAATTAACGATGAGAGAATT	Downstream homology for pTB4
20	ComECKO_down_B	CTCGAGTTTTTCAGCAAGATCTAAACGCCACGGTAAGAACG	
21	pilA1KO_up_A	ATTGTAGGAGATCTTCTAGAAAGATGGCCCTGCAACAGTTAAGGG	Upstream homology for pTB5
22	pilA1KO_up_B	AACCTCTTACGTGCCGATCAGATTGTCTTCTTCCTTCTGTAGGGG	
23	pilA1KO_down_A	AGTGGCAGGGCGGGGCGTAAGACCCTATTATGTTTTGAGTGGTGC	Downstream homology for pTB5
24	pilA1KO_down_B	CTCGAGTTTTTCAGCAAGATCCTTTCGCTCTCCACACATAGT	
25	pilQKO_up_A	ATTGTAGGAGATCTTCTAGAAAGATGCGTCTTGGATCGACCTGC	Upstream homology for pTB6
26	pilQKO_up_B	AACCTCTTACGTGCCGATCAGACCGTTAGCGATAATCTACCAGC	
27	pilQKO_down_A	AGTGGCAGGGCGGGGCGTAATTAAATTCCTAGCGGGAGACTAAAGTG	Downstream homology for pTB6
28	pilQKO_down_B	CTCGAGTTTTTCAGCAAGATTACAGGGTTACGAAGACCGGG	
29	RecJ_a1	CCGTTCCGTGGCAAAGCAAAATTGCAGAAGAACAATTCCAAAAGC	Upstream homology for pJET-Δ*recJ*-*nptI*
30	RecJ_a2	CTCGAGTTTTTCAGCAAGATTCCCTTGGATGCACACAAAATATCG	
31	nptI_fwd	GTGTCTCAAAATCTCTGATGTTACATTG	nptI gene
32	nptI_rev	TTTGCTTTGCCACGGAACG	
33	RecJ_b1	ACATCAGAGATTTTGAGACACGGTTAGAGAATTGAAAGGGATTCCATACT	Downstream homology for pJET-Δ*recJ*-*nptI*
34	RecJ_b2	TGTAGGAGATCTTCTAGAAAGATCAATAGCTTTGAGGGCTTTATTAATGC	
35	1-f	TTAGCAATTGTGCAATGCTGGAGAG	

#	Given name	Sequence 5' -> 3'	Purpose
36	1-r	AACCTGGAAAATGATGGGAGCC	Detection of pCA and pCA2.4
37	2-f	GGAGTTTCTGGTATTTTCTAGCAGTATGCC	Detection of pCB and pCB2.4
38	2-r	CAAATTTGACTAATAGTTTTGATATTTTATTACTAAAAC	
39	3-f	TACAAGTCAATCACCGCAAGGATTCTAG	Detection of pCC5.2
40	3-r	GCTTGCCTGCAAAATGTGCG	
41	27F1	AGAGTTTGATCCTGGCTCAG	16S rRNA control
42	1494Rc	TACGGCTACCTTGTTACGAC	
